# Chest wall volume and asynchrony in stroke and Parkinson’s disease subjects: A case-control study

**DOI:** 10.1371/journal.pone.0216641

**Published:** 2019-05-16

**Authors:** Rêncio Bento Florêncio, Antonio José Sarmento da Nobrega, Íllia Nadinne Dantas Florentino Lima, Lucien Peroni Gualdi, Elis Emmanuelle Cabral, Marina Lyra Lima Cabral Fagundes, Andrea Aliverti, Vanessa Regiane Resqueti, Guilherme Augusto de Freitas Fregonezi

**Affiliations:** 1 PneumoCardioVascular Laboratory, Hospital Universitário Onofre Lopes, Empresa Brasileira de Serviços Hospitalares (EBSERH), Departamento de Fisioterapia, Universidade Federal do Rio Grande do Norte, Natal, Brazil; 2 Laboratório de Inovação Tecnológica em Reabilitação, Departamento de Fisioterapia, Universidade Federal do Rio Grande do Norte, Natal, Rio Grande do Norte, Natal, Brazil; 3 Faculdade de Ciências da Saúde do Trairi, Federal University of Rio Grande do Norte, Santa Cruz, Rio Grande do Norte, Brazil; 4 Dipartimento di Elettronica, Informazione e Bioingegneria, Politecnico di Milano, Milano, Italy; Vanderbilt University Medical Center, UNITED STATES

## Abstract

**Background:**

The expansion of the rib cage and abdomen occurs in a synchronic way during a coordinated contraction of the diaphragm and the abdominal and intercostal muscles under normal conditions and healthy. The presence of restrictive respiratory disease may lead to uncoordinated action of the respiratory muscles which affects breathing pattern and chest wall volumes. The aim of this study was to evaluate chest wall volumes, chest wall asynchrony and inspiratory paradoxical movement of breathing, as well as the influence of the time of disease diagnosis in subjects with Parkinson’s disease and post-Stroke in comparison to healthy individuals.

**Methods:**

Total and compartmental chest wall volumes, chest wall asynchrony and paradoxical movement were measured at rest in a seated position by Optoelectronic Plethysmography in 76 individuals (29 healthy individuals, 20 post-Stroke and 27 Parkinson’s disease subjects). Post-stroke and Parkinson’s disease subjects were also grouped according to the length of diagnosis.

**Results:**

In both groups with restrictive respiratory disease we observed that pulmonary rib cage compartment (V_RCp_) volume is reduced when compared to healthy subjects (p <0.05). This same pattern was observed when analyzing post-stroke subjects with more than three years of diagnosis and Parkinson’s subjects with less than three years of diagnosis (p<0.05). Furthermore, post-stroke subjects with inspiratory paradoxical movement showed decreased total and compartmental chest wall volumes (p<0.05), while individuals with Parkinson’s disease with inspiratory paradoxical movement only presented a decrease in pulmonary rib cage compartment volume (p<0.05).

**Conclusion:**

Our study presents new findings for better understanding of chest wall volumes and chest wall asynchrony in post-stroke and Parkinson’s disease individuals. Half of the subjects with post-Stroke and Parkinson’s disease presented inspiratory paradox movement, but changes in breathing pattern was especially observed in post-stroke subjects with more than three years of diagnosis.

## Introduction

Ventilation is a complex process determined by diaphragm, rib cage and abdominal muscle contraction which results in changes in airway pressures and chest wall volumes [1]. In the past, the three-physiological compartment model of the chest wall that includes Pulmonary Rib Cage (RCp), Abdominal Rib Cage (RCa) and Abdomen (AB) was recognized as a model that could explain the contribution of all respiratory muscles in the chest wall volume, pressure and flow changes [2].

Expansion of the rib cage and abdomen in healthy subjects occurs in a synchronic way during a coordinated contraction of the diaphragm, abdominal and intercostal muscles [3]. However, the presence of restrictive respiratory disease may lead to uncoordinated action of the respiratory muscles which affects the breathing pattern and chest wall volumes. Previous studies conducted by our group showed significant impairment in breathing pattern in Parkinson’s disease and post-stroke subjects at rest and during the use of Positive Expiratory Pressure [4,5]. Furthermore, we and other authors have observed an asymmetry in chest wall volume variation in post-stroke subjects [5,6].

Respiratory muscle dysfunction is present in varying degrees in Parkinson’s disease and post-stroke subjects [7–11]. Although forced expiratory volume in the 1^st^ second (FEV_1_) and forced vital capacity (FVC) provide sufficient data to diagnose restrictive respiratory pattern, it is a static measurement that cannot provide precise information about breathing pattern and dynamic chest wall volumes changes. Studies of dynamic chest wall volumes through optoelectronic measurement were described some years ago. Optoelectronic Plethysmography (OEP) is a reliable method for analyzing chest wall kinematics and its compartments [12–14]. Using OEP it is possible to accurately analyze the delay between chest wall compartment movements, defined as chest wall asynchrony. Thus, chest wall asynchrony occurs when there is a delay in a compartment—RCp, RCa or AB—in relation to another, as well as the presence of inspiratory paradoxical movement which occurs when the compartments move in opposite directions during inspiration [15, 16]. Inspiratory paradoxical movement was recently defined considering data from phase shift angle derived from Lissajous figures that provides the degree of synchrony and inspiratory paradox time, which is the fraction of inspiratory time relative to the chest wall volumes, in which compartmental volume decreases [17, 18].

The mechanisms underlying the presence of chest wall asynchrony, inspiratory paradoxical motion and the relationship with disease time in Parkinson’s disease and post-stroke subjects are not fully understood. Some studies have already been described in the literature [19–23] for evaluating several pathological conditions through the OEP, including hemiparetic subjects and Parkinson’s disease. However, chest wall volumes, chest wall asynchrony and inspiratory paradoxical motion have never been studied in detail through Optoelectronic Plethysmography in these populations, when compared to healthy.

Thus, the aim of our study was to analyze chest wall volumes, chest wall asynchrony and inspiratory paradoxical movement of breathing, as well as the influence of disease time diagnosis in Parkinson’s disease and post-stroke subjects.

## Material and methods

### Subjects

Adult subjects with Parkinson’s disease, post-stroke subjects and healthy individuals were recruited for the study. Individuals with Parkinson’s diagnosis in stages II and III according to the Hoehn and Yahr scale, without cognitive changes due to the effects of Levodopa were recruited [24]. Hemiparetic subjects with stroke diagnosis confirmed by CT scan, with an interval of at least 6 months since the event and preserved cognition were also recruited. Parkinson’s subjects were assessed in ON condition (under the effect of levodopa therapy). Self-reported healthy individuals matched for age and gender for both disease groups were recruited for the control group. Those individuals who could not remain seated during the protocol or quit the study during data collection and showing pulmonary and/or cardiac diseases were excluded. The study was approved by the Research Ethics Committee of the University Hospital (protocol no. 095/ 2011). All participants provided written informed consent in accordance with Declaration of Helsinki.

### Study design

After assessment of anthropometric characteristics, pulmonary function and respiratory muscle strength tests, the subjects were asked to sit in a chair where the 89 retroreflective markers were placed. A previously trained examiner subsequently performed the placement of 89 reflective markers on subjects’ trunk to capture chest wall kinematics by OEP. Subjects remained seated for 180 seconds in quiet breathing for data acquisition.

### Measurements

#### Pulmonary function and respiratory muscle strength

Pulmonary function testing was assessed using a spirometer (Koko DigiDoser, Spire, Longmont, USA) following the technical procedures recommended by ATS/ERS [25], and predicted values were calculated using previously published reference values for the study population [26]. Absolute and percentage of predicted values of forced vital capacity (FVC), forced expiratory volume in the first second (FEV_1_) and FEV_1_/FVC ratio were considered.

Respiratory muscle strength was assessed by measuring maximal respiratory pressures [maximal inspiratory pressure (MIP) and maximal expiratory pressure (MEP)] using a MicroRPM manovacuometer (Micro Medical, Rochester, Kent, UK), following previously described procedures [27], and standardized reference values ​​for the studied population were used to calculate predicted values [28]. The tests were performed with subjects in the sitting position and from residual volume (RV).

#### Optoelectronic plethysmography

Chest wall (CW) kinematics and its compartments (pulmonary ribcage [RCp], abdominal ribcage [RCa] and AB) were assessed using optoelectronic plethysmography (OEP System; BTS Engineering, Milan, Italy) [14]. First, 89 retroreflective markers were placed on specific anatomic points of the thorax and abdomen of the patients, and six previously-calibrated TV cameras captured the marker’s position at a frequency of 60 frames·sec^-1^ while the subjects remained seated on a chair breathing quietly for 180 seconds. The time-courses of the volume of each compartment (V_RCp_, V_RCa_ and V_AB_) along with their sum (V_CW_) were computed by means of an algorithm based on the Gauss’ theorem and processed to obtain a breath-by-breath assessment of both ventilatory pattern (breathing frequency [f], inspiratory time [Ti], expiratory time [Te] and minute ventilation [VE]) and chest wall volumes [29]. All measurements of the respiratory function were performed at the same time during OEP records, for all individuals in the present study.

#### Chest wall asynchrony and paradoxical motion

**Fig 1:**
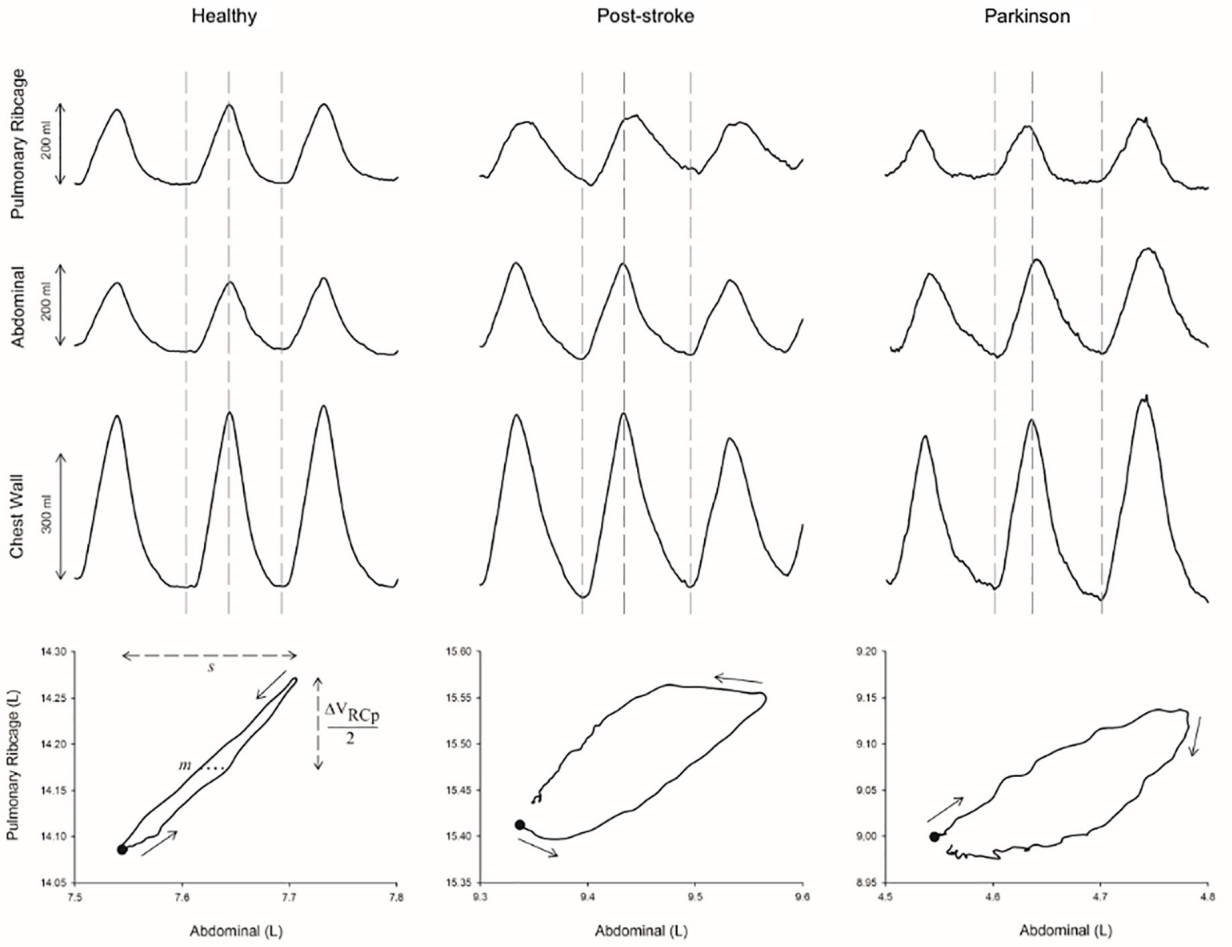
Graphical representation of the phase angle calculation using the Lissajous Curve. ΔV_RCp_: upper RC variation; m: 50% of ΔV_RCp_; s: maximal excursion of abdominal compartment (L).

The degree of asynchrony between AB and RCp compartments was calculated after the construction of Lissajou loops, as previously described [17]. The phase angle (θ), indicated by the degree of opening of the Lissajou figure produced when these two volumetric signals were plotted against each other, was defined by the following formula: θ = sin^-1^ (m/s), where *m* represents the distance delimited by the intercepts of the dynamic loop on a line parallel to the X-axis (V_AB_) at 50% of the tidal volume of the signal on the Y-axis (V_RCp_), and *s* represents the tidal volume of the signal on the X-axis (Fig 1). In this system, a phase angle of zero represents completely synchronous movement of the compartments and 180° total asynchrony. In addition, a positive θ means that RCp is leading AB expansion and a negative angle describes the reverse situation [17, 18].

The inspiratory paradox time of the RCp (IP_RCp_), defined as the fraction of the inspiratory time in which the volume of the RCp compartment decreases relative to the total V_CW_ and expressed as percentage, was used to define paradoxical rib cage movement, as a way of reinforcing the subjects who present asynchrony confirmed by the phase angle (θ) [17]. For data analysis, median as well as 25-75^th^ interquartile range data of healthy subjects were used to define the normal ranges of θ and IP, as well as to classify the subjects as patients with (P+) or without (P-) inspiratory paradoxical movement of the RCp during quiet breathing. Threshold values of θ and IP were obtained during a period of ~ 50 respiratory cycles at rest, and P+ was defined when both θ and IP values were beyond the upper and lower limits of control data [17].

### Statistical analyses

Data are shown in mean ± SD or median [interquartile range 25–75%] according to distribution. Parkinson’s disease and post-stroke subjects were divided into two groups according to length of disease diagnosis time as: 1) less than 3 years (< 3); and 2) equal to or more than three years (≥ 3), in order to evaluate if the disease time negatively influenced lung function and/or volume pattern in individuals. Data distribution was analyzed using the *Kolmogorov-Sminorv* test. Differences between groups were analyzed using the One-way ANOVA or Kruskal-Wallis test. In the event of statistically significant differences, Bonferroni’s or Dunns *post-hoc* tests were applied to identify the difference between groups. The Mann-Whitney test was used to compare subgroups of Parkinson’s disease and post-stroke subjects with and without paradoxical movement. Effect-sizes were calculated using G*Power software (G*Power 3.1.9.2, Kiel, Germany). Cohen’s f was also expressed (<0.20) as moderate (0.25 and 0.40) or large (>0.40) [30]. Inferential analyses were performed using GraphPadPrism 5.0 (La Jolla, Ca, USA). A significance level of 5% (p <0.05) was adopted for all statistical analyses.

## Results

We studied 28 subjects with Parkinson’s disease, 23 post-stroke subjects and 36 healthy individuals. One subject from the Parkinson’s disease group, 3 from the post-stroke group and 7 healthy subjects were excluded from the study due to irregularities and artifacts originated in data collection. The final sample size was composed of 27 subjects with Parkinson’s disease, 20 post-stroke individuals, and 29 healthy adults matched for age and gender. A post hoc analysis considering the calculated effect-size for IP_Rcp_ between groups (Cohen’s *f* = 1.9) showed a statistical power (1-β) = 0.97 for this study.

### Anthropometric characteristics, pulmonary function and respiratory muscle strength

No significant differences were found regarding anthropometric characteristics among the groups for age, weight, height and body mass index, as shown in Table 1.

**Table 1:** Anthropometric characteristics, predicted values of lung function and respiratory muscle strength for healthy individuals, subjects with Parkinson’s disease and post-stroke subjects according to length of diagnosis time.

				Post-stroke		Parkinson	
Subjects _(n)_	Healthy	Post-stroke	Parkinson	< 3	≥ 3	< 3	≥ 3
	29	20	27	13	7	8	19
Age _(years)_	57 [51.5–63,5]	57 [52.2–62.5]	61.5 [54.5–67.7]	56 [49.5–61]	58 [55–68]	61 [47.2–67.5]	62.5 [58.2–67.7]
BMI _(Kg/m_^2^_)_	26 [24.1–28.5]	25 [23–29]	25.8 [23.1–28.45]	24 [22.5–28.5]	28.4 [25.2–30.3]	26.2 [24.1–27.3]	24.9 [21.8–28.9]
FVC _(L)_	3.83 [3.68–4.34]	3.30 [2.76–3.60] ^#^	3.2 [2.54–3.81] ^#^	3.26 [2.28–3.40] ^#^	3.64 [3.2–3.81]	2.98 [2.51–3.22] ^#^	3.50 [2.60–3.90]
FVC _(%pred)_	95 [89.9–100]	82 [76.5–98.2]	86.5 [75.5–94.4]	82 [77–96.5]	89.6 [78–111.3]	76.5 [69.5–91.8] ^#^	88.1 [82.5–95.7]
FEV_1 (L)_	3.25 [2.87–3.55]	2.72 [2.05–2.97] ^#^	2.70 [1.97–3.07] ^#^	2.74 [1.81–2.86] ^#^	2.70 [2.25–3.10]	2.55 [2.03–2.73] ^#^	2.74 [1.92–3.10] ^#^
FEV_1 (%pred)_	95 [88.4–99.5]	82 [76.5–99]	86 [78.5–96.5]	80 [73–95.5]	82.9 [80.8–101]	79 [73.7–85.5]	91.3 [81.6–97.5]
FEV_1_ /FVC	0.80 [0.78–0.84]	0.81 [0.76–0.85]	0.82 [0.76–0.85]	0.82 [0.79–0.84]	0.78 [0.70–0.85]	0.84 [0.82–0.91]	0.75 [0.67–0.87]
FEV_1_ /FVC _(%pred)_	100 [96.6–103]	99.5 [92.5–103]	89.7 [79.7–103.5]	100 [94–103.4]	99 [91.2–106.8]	103.7 [100–110.1]	80.8 [78.2–97.1] ^#^
MIP _(cmH2O)_	107 [96.5–132.5]	66.5 [54.5–97.5] ^#^	72 [57–102] ^#^	65 [56–80] ^#^	71 [53–110]	81.5 [52.5–109.8]	69 [57–102] ^#^
MIP _(%pred)_	104 [91.2–124.2]	68.5 [59.2–85] ^#^	74.1 [58.5–98.4] ^#^	67 [60.5–82.5] ^#^	70.9 [58.1–101]	77 [63.3–98]	68.7 [57.2–93.5] ^#^
MEP _(cmH2O)_	124 [109–161.5]	88 [72–119.5] ^#^	97 [81–122] ^#^	81 [69–96.5] ^#^	118 [79–122]	109 [76.5–131]	97 [81–116] ^#^
MEP _(%pred)_	116.9 [102–141.3]	88.5 [75.2–108] ^#^	99.5 [80.2–108.9] ^#^	88 [75.5–103]	103.4 [71.2–122.7]	96.3 [74.5–108]	99.5 [81.6–111.1]

Data presented as median and interquartile range between 25–75%. FVC: Forced Vital Capacity; FEV_1_: Forced expiratory volume in the 1^st^ second; FEV_1_/FVC: Forced expiratory volume in the first second/forced vital capacity ratio; MIP: Maximum inspiratory pressure; MEP: Maximum expiratory pressure; n: number of subjects; m: meters; kg: kilograms; L: Liters; %pred: Percentage of predicted; non-parametric data distribution;

^#^ <0.05 *versus* Healthy.

Parkinson’s disease and post-stroke subjects showed significantly lower absolute values for FVC and FEV_1_ when compared to healthy controls (p < 0.05). When the subgroups were compared with healthy controls, significantly lower absolute values of FVC and FEV_1_ were observed in <3 post-stroke subjects. On the other hand, Parkinson subjects with < 3 years of diagnosis also presented lower values (p < 0.05) of FVC and FEV_1_ in absolute values, and FVC_%pred_ when compared with healthy controls. Furthermore, a significantly lower FEV_1_/FVC_%pred_ (p < 0.05) was observed when Parkinson’s subjects with ≥ 3 years of diagnosis were compared to healthy controls.

Regarding respiratory muscle strength, Parkinson’s and post-stroke subjects showed significantly lower values for maximal inspiratory pressure (p < 0.05) and maximal expiratory pressure (p < 0.05) when compared to healthy individuals. When subgroups were analyzed, those post-stroke subjects with <3 years of diagnosis and those subjects with Parkinson’s disease with ≥ 3 of diagnosis presented lower values for MIP and MEP, respectively, when compared to healthy controls (p < 0.05) (Table 1).

### Breathing pattern and chest wall volumes

Regarding V_CW_, no differences were observed between groups. When the compartmental chest wall volume (V_RCp_, V_RCa_ and V_AB_) analysis was performed, both post-stroke and Parkinson’s subjects showed significantly lower values of V_RCp_ when compared to healthy controls (p < 0.05). Moreover, the V_RCa_ of post-stroke subjects was also significantly lower when compared with healthy controls [0.06L (0.04–0.10) vs. 0.10L (0.08–0.16), respectively (p < 0.01) (Table 2).

**Table 2:** Breathing pattern at rest of healthy individuals and Parkinson’s disease and post-stroke subjects according to the length of disease diagnosis.

				Post-stroke		Parkinson	
Variables	Healthy	Post-stroke	Parkinson	< 3	≥ 3	< 3	≥ 3
V_CW (L)_	0.518 [0.428–0.793]	0.468 [0.392–0.558]	0.512 [0.394–0.738]	0.483 [0.393–0.583]	0.450 [0.386–0.537]	0.436 [0.376–0.559]	0.539 [0.433–0.752]
V_RCp (L)_	0.183 [0.154–0.276]	0.135 [0.097–0.15] ^#^	0.125 [0.084–0.194] ^#^	0.150 [0.111–0.201]	0.061[0.046–0.129] ^#^	0.103 [0.075–0.121] ^#^	0.169 [0.094–0.205]
V_RCa (L)_	0.099 [0.081–0.155]	0.065 [0.043–0.10] ^#^	0.083 [0.050–0.170]	0.073 [0.045–0.115]	0.062 [0.036–0.074] ^#^	0.070 [0.050–0.157]	0.090 [0.055–0.176]
V_AB (L)_	0.224 [0.181–0.380]	0.254 [0.178–0.389]	0.300 [0.197–0.437]	0.185 [0.143–0.381]	0.299 [0.221–0.407]	0.272 [0.201–0.327]	0.300 [0.197–0.456]
Ti _(s)_	1.65 [1.4–2.26]	1.61 [1.366–2.763]	1.70 [1.40–2.41]	1.44 [1.29–1.86]	1.33 [1.09–1.43]	1.32 [1.00–1.62]	1.45 [1.02–1.75]
Te _(s)_	2.60 [2.20–3.20]	2.07 [1.63–2.80]	2.33 [1.88–3.26]	2.18 [2.01–3.07]	1.60 [1.21–1.71] ^#^	1.78 [1.59–2.80]	1.69 [1.48–2.65]
Ttot _(s)_	4.26 [3.85–5.75]	3.45 [2.95–4.27]	4.24 [3.43–5.47]	3.70 [3.35–4.76]	2.822 [2.46–3.00] ^#^	3.03 [2.62–4.62]	4.35 [3.81–5.93]
*f*_(bpm_^-1^_)_	14.3 [11.5–15.8]	17.9 [14.3–20.4]	14.2 [11–17.4]	16.2 [13.8–18.5]	21.6 [20.2–24.6] ^#^	19.9 [13.5–23]	13.93 [10.5–16.1]
VE _(L/min_^-1^_)_	7.87 [6.36–9.03]	8.28 [6.13–9.99]	7.85 [6.59–9.42]	7.74 [6–8.82]	11.1 [6.97–12.9]	8.7 [7.27–9.58]	7.48 [6.57–8.51]
Phase Angle (°)	4.98 [2.67–9.74]	11.91 [5.78–19.05] ^#^	6.77 [3.38–20.30]	11.98 [5.85–18.61]	15.33 [8.29–29.34]	6.37 [4.17–15.68]	6.77 [3.12–24.76]
IP_RCp_ (%)	19.44 [15.74–26.55]	29.40 [23.20–36.78] ^#^	29.28 [14.62–34.58]	24.86 [21.64–31.97]	35.47 [34.08–41.98] ^#^	29.85 [11.70–35.82]	26.79 [14.44–33.33]

Data presented as median and interquartile range between 25–75%.; V_CW_: Chest wall volume; V_RCp_: Pulmonary ribcage volume; V_RCa_: Abdominal ribcage volume; V_AB_: Abdominal volume; Ti: Inspiratory time; Te: Expiratory time; Ttot: Total time of the respiratory cycle; *f*: breathing frequency; VE: Minute volume; IP_RCp_ (%): Inspiratory paradox time in percentage; L: Liters; min: minutes; s: seconds; Bpm: Breaths per minute; non-parametric data distribution;

^#^ <0.05 *versus* Healthy.

Parkinson’s subjects with <3 years of diagnosis and post-stroke subjects with ≥ 3 years diagnosis showed lower volumes in RCp and RCa compartments, respectively, in comparison to healthy controls (p < 0.05), as shown in Table 2.

Expiratory time (Te) was significantly lower (p < 0.05) in post-stroke subjects with three or more years of diagnosis in comparison to healthy individuals (Table 2). Regarding breathing frequency (*f*), significantly higher values were observed in ≥ 3 years of diagnosis post-stroke group in comparison to healthy controls (p < 0.05), as shown in Table 2. Finally, total time of respiratory cycle was statistically different between post-stroke subjects with 3 or more years of diagnosis and healthy individuals (p < 0.05, Table 2).

**Fig 2:**
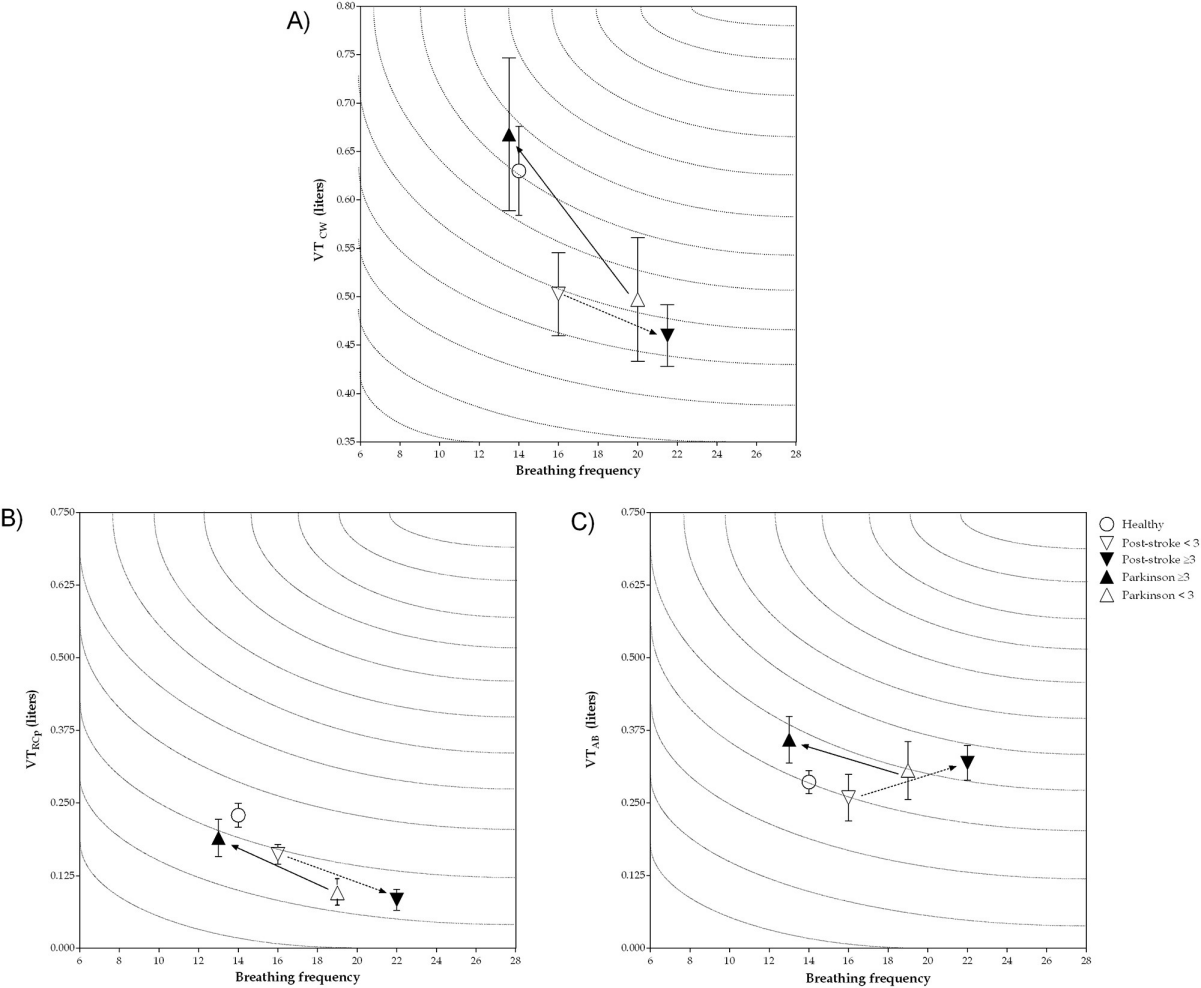
Minute ventilation of chest wall and rib cage pulmonary and abdomen compartments at rest in healthy individuals, post-stroke and Parkinson’s disease subjects according to length of disease diagnosis. Data presented as median and interquartile range between 25–75%. A) minute ventilation of chest wall; B) minute ventilation of rib cage pulmonary compartment; C) minute ventilation of abdomen compartment; VT_CW_: tidal volume of chest wall (in liters); VT_RCp_: tidal volume of rib cage pulmonary compartment (in liters); VT_AB_: tidal volume of abdomen compartment (in liters).

VE did not show any significant difference among the groups; however, a characteristic pattern was observed when plotting minute ventilation considering V_RCp_ *versus* the length of diagnosis time. Post-stroke subjects with less than three years of diagnosis showed decreased lung volumes (for chest wall, pulmonary rib cage and abdominal) and higher breathing frequency when compared to healthy subjects. Such volume reduction and increased breathing frequency is even more evident in the group with 3 or more years of diagnosis as shown in Fig 2, although there is no significant statistical difference.

### Chest wall asynchrony and paradoxical movement

As shown in Table 2, a significant difference (p < 0.05) was observed regarding θ only between post-stroke and healthy subjects. When subjects were analyzed according to diagnosis time, post-stroke subjects presented greater asynchrony, without a significant difference and independent of the disease time. This same pattern was not observed in the subjects with Parkinson’s disease for less than 3 years or with 3 or more years of diagnosis, as the phase angle was close to healthy controls (Table 2). Regarding IP_RCp_, significant differences were found when we compared all post-stroke individuals (p < 0.05), as well as those grouped as ≥ 3 years of diagnosis (p < 0.001) in comparison to healthy controls (Table 2).

### Paradoxical ribcage movement

Table 3 shows anthropometric characteristics, lung function, respiratory muscle strength and breathing pattern in subjects with paradoxical movement, considering the θ and IP variables together. All individuals of post-stroke and Parkinson’s disease groups were subdivided among those who presented paradoxical movement (P+) and those who did not (P-), based on the threshold values for phase angle and IP_RCp_ of the healthy group who were described in the 25-75^th^ percentile of 2.67 to 9.74° and 15.74 to 26.55%, respectively. The individuals who were in the lower or upper limit of both thresholds were considered as having paradoxical movement.

**Table 3:** Anthropometric characteristics, lung function, respiratory muscle strength, breathing pattern and chest wall asynchrony at rest for post-stroke and Parkinson’s disease subjects according to paradoxical movement.

Variables	Post-stroke		Parkinson’s disease	
	P -	P +	P -	P +
Subjects _(n / %)_	9 [45%]	11 [55%]	12 [44.5%]	15 [55.5%]
< 3 _(disease time)_	8 [61.5%]	5 [38.5%]	3 [37.5%]	5 [62.5%]
≥ 3 _(disease time)_	1 [15%]	6 [85%]	9 [47.4%]	10 [52.6%]
Age _(years)_	53 [49.5–63]	58 [55–63]	63 [59.5–67]	59 [51–69]
BMI _(kg/m_^2^_)_	24 [22.5–29]	25.6 [23.8–30.1]	27.6 [21.8–30.6]	24.9 [23.3–26.7]
FVC _(L)_	2.94 [2.12–3.47]	3.42 [3.20–3.75]	3.34 [2.52–3.97]	3.10 [2.54–3.80]
FVC _(% pred)_	79 [74–87.5]	89.6 [80–111.3]	87.4 [82.5–100.1]	87 [69–94.4]
FEV_1 (L)_	2.28 [1.74–2.94]	2.74 [2.25–2.98]	2.63 [1.92–3.15]	2.74 [1.97–3.0]
FEV_1 (% pred)_	80 [69.5–83.9]	87 [80–104]	92.8 [86–102.4]	80.6 [73–91] *
FEV_1_/FVC	0.80 [0.75–0.84]	0.81 [0.76–0.86]	0.81 [0.76–0.85]	0.82 [0.75–0.85]
FEV_1_/FVC _(%pred)_	100 [90.5–104]	99 [99.5–103.9]	84.8 [78.2–102.3]	90.5 [80.7–103.8]
MIP _(cmH2O)_	76 [62–116.5]	61 [53–71]	69 [57.5–92.8]	76 [49–113]
MIP _(%pred)_	82 [65–100.6]	66 [43–71]	80.9 [67.1–111.2]	66.4 [58–100]
MEP _(cmH2O)_	72 [66–104]	93 [79–122]	102.5 [80.5–125]	93 [81–116]
MEP _(%pred)_	87.8 [76.5–95]	103.4 [71.2–115]	107.2 [100.7–114.8]	86 [74.4–100] *
V_CW (L)_	0.454 [0.393–0.583]	0.503 [0.386–0.548]	0.486 [0.38–0.81]	0.512 [0.44–0.66]
V_RCp (L)_	0.150 [0.12–0.24]	0.118 [0.05–0.14] *	0.180 [0.12–0.36]	0.095 [0.04–0.12] *
V_RCa (L)_	0.093 [0.07–0.13]	0.045 [0.03–0.07] *	0.086 [0.05–0.18]	0.081 [0.04–0.17]
V_AB (L)_	0.184 [0.13–0.28]	0.344 [0.21–0.41] *	0.252 [0.17–0.44]	0.333 [0.27–0.43]
Ti _(s)_	1.33 [1.18–1.90]	1.39 [1.30–1.52]	2.13 [1.21–2.64]	1.58 [1.40–2.26]
Te _(s)_	2.03 [1.83–3.21]	2.12 [1.60–2.86]	2.90 [1.61–4.25]	2.29 [2.02–2.56]
Ttot _(s)_	3.61 [3.09–5.05]	3.41 [2.82–4.28]	4.91 [2.73–6.81]	3.84 [3.46–4.98]
*f*_(bpm_^-1^_)_	17.35 [12.60–19.62]	18.49 [14.42–21.60]	12.52 [9.13–22.33]	15.75 [12.45–17.41]
VE _(L/min_^-1^_)_	7.743 [6.23–9.12]	8.669 [6.02–11.16]	7.501 [5.80–8.44]	8.247 [6.70–9.44]

Data presented as median and interquartile range between 25–75%. P+: individuals with paradoxical movement; P-: individuals without paradoxical movement; n: number of subjects; < 3 (disease time): individuals less than three years of disease time; ≥ 3 (disease time): individuals equal to or more than three years of disease time; BMI: body mass index; FVC: Forced Vital Capacity; FEV_1_: Forced expiratory volume in the 1^st^ second; FEV_1_/FVC: Forced expiratory volume in the first second/forced vital capacity ratio; MIP: Maximum inspiratory pressure; MEP: Maximum expiratory pressure; m: meters; kg: kilograms; L: Liters; %pred: Percentage of predicted; V_CW_: Chest wall volume; V_RCp_: Pulmonary rib cage volume; V_RCa_: Abdominal ribcage volume; V_AB_: Abdominal volume; Ti: Inspiratory time; Te: Expiratory time; Ttot: Total time of the respiratory cycle; *f*: breathing frequency; Bpm: Breaths per minute; VE: Minute volume; L: Liters; min: minutes; s: seconds; non-parametric data distribution; Mann-Whitney test;

* p < 0.05 for comparison of P+ with P-.

When all post-stroke subjects were analyzed, it was found that 11 of 20 individuals (55%) presented paradoxical movement (P+). In addition, 38.5% of the subjects with < 3 years of diagnosis and 85% of with ≥ 3 years of diagnosis presented P+. Among the Parkinson’s disease group, 50 subjects (55.5%) met both criteria for paradoxical movement (P+). Moreover, when considering the diagnosis time, it was observed that 62.5% among individuals with less than three years of diagnosis showed paradoxical movement, while 52.6% among those with 3 or more years of diagnosis were P+. FEV_1_ in predicted percentage presented significantly higher values for P- individuals when compared to P+ individuals in Parkinson’s disease (p < 0.05), as well as for maximal expiratory pressure in predicted percentage (p < 0.05).

Regarding chest wall volumes, it was observed that P+ individuals (for Parkinson’s disease as well as post-stroke subjects) had lower values for pulmonary rib cage (V_RCp_) tidal volume when compared to P-. Only P- post-stroke subjects presented a significant difference when analyzing abdominal rib cage tidal volume (V_RCa_) with higher values (p < 0.05), and abdominal tidal volume (V_AB_) with lower values (p < 0.05) compared to P+ individuals. Post-stroke subjects as well as Parkinson’s disease subjects with paradoxical movement presented higher values for the phase angle and IP_RCp_, as expected.

## Discussion

In this study, we aimed to describe a detailed analysis of chest wall volumes, chest wall asynchrony and inspiratory paradoxical movement using OEP in post-stroke and Parkinson’s subjects, as well as to compare them to healthy controls. The OEP is a non-invasive, reliable and precise device that measures chest wall volume and is capable of detecting small movements during breath-by-breath based on optical technology. Moreover, OEP is highly accurate in measuring total chest wall volume variations, allowing for dividing the complex shape of the chest wall into different compartments [17, 18]. The main results of the present study were: 1) the pulmonary rib cage compartment volume is reduced in both groups when compared to healthy subjects, and this pattern was also observed in post-stroke subjects with more than three years of diagnosis and Parkinson’s subjects with less than three years of diagnosis; 2) the breathing pattern became less efficient in post-stroke subjects with more than three years of diagnosis, whereas there is a tendency to improve respiratory rate and pulmonary rib cage compartment volume in Parkinson’s subjects with more than three years of diagnosis (Fig 2); and 3) post-stroke subjects with inspiratory paradoxical movement showed decreased total and compartmental chest wall volumes, while individuals with Parkinson’s disease with inspiratory paradoxical movement only showed a decrease in pulmonary rib cage compartment volume.

In post-stroke subjects, changes in breathing pattern and chest wall volumes may potentially result in lower ventilatory efficiency with progressive inefficiency of respiratory muscles leading to the development of restrictive respiratory pattern [31, 32]. Moreover, disease progression leads to a spiral restrictive respiratory pattern, increased chest wall loads and the conversion of muscle contractions into chest wall expansion becomes progressively more difficult. On the other hand, respiratory dysfunction in subjects with Parkinson’s disease was described by Sir James Parkinson in 1817 [33]. The restrictive respiratory pattern in Parkinson’s disease is not fully understood [20] due to its relation to several factors such as abnormal activity of accessory respiratory muscles [34, 35], abnormal ventilatory control [20], increased chest wall rigidity and decreased lung volume due to kyphoscoliosis.

Our findings are the first to demonstrate via OEP that even patients without important respiratory restrictive pattern showed decreased pulmonary rib cage compartment volume in both groups. Moreover, breathing pattern was impaired, especially in post-stroke subjects with more than three years of diagnosis. In these subjects, we observed an increase in minute ventilation associated with decreased pulmonary rib cage volume, further related to an abnormal increase in respiratory rate and a decrease in inspiratory and expiratory time when compared to healthy subjects. Although predicted values of FVC and FEV_1_ are slightly below normal, maximal inspiratory pressure is moderately reduced. Breathing pattern and respiratory muscle strength were previously investigated by Teixeira-Salmela et al. [22]. In a case control study with 16 post-stroke subjects, these authors found lower tidal volume of around 255 ml, a higher respiratory rate of 17.36 bpm^-1^ and similar respiratory muscle strength when compared to our study. Despite the methodological quality of the results published by Teixeira-Salmela et al. [22], the respiratory inductance plethysmography technique used to study breathing pattern has reduced accuracy and limited validity. The assessment of volume changes derived by changes in diameter or cross-sectional area of a single transverse section are problematic for both the rib cage and the abdomen and measuring changes in chest wall volume is subject to different errors [36]. Indeed, respiratory inductance plethysmography is a technique with limited use in clinical and research fields.

Regarding Parkinson’s disease, decreased pulmonary rib cage compartment volume was observed in all group subjects, as well as when they were grouped into less than three years of diagnosis. However, our data found by the small number of patients when we grouped individuals by time of diagnosis is consistent with the literature. Treatment of Parkinson’s disease with levodopa can lead to a significant improvement in respiratory function. Despite controversial literature regarding the effects of levodopa on restrictive respiratory pattern, some studies have shown an improvement in respiratory function after treatment with levodopa [37–39]. In fact, in our study we found an improvement of breathing efficiency in patients with more than three years of diagnosis, with a decrease in respiratory rate and an increase in tidal volume (Fig 2). Moreover, no significant difference was found in pulmonary rib cage compartment volume in these groups of patients. A previous study performed by Vercueil et al. [21] assessing breathing pattern through respiratory inductance plethysmography during ON condition showed similar a breathing pattern to our results. A small discrepancy with the present study in the variables of tidal volume, minute ventilation, inspiratory and expiratory time can be explained by different techniques used by the studies and the duration of the disease in the group studied by those authors. Further studies should be performed regarding breathing pattern data in subjects with Parkinson’s disease to confirm our findings.

The technique to measure chest wall asynchrony and paradoxical movement using optoelectronic plethysmography was recently described [17, 18]. The asynchrony between the rib cage and abdominal muscles was related to failure during mechanical ventilation weaning [40], in other words it can be related to respiratory system inefficiency. On the other hand, we adopted a previously described analyses of inspiratory paradoxical chest wall movement to improve understanding of chest wall asynchrony pattern. Paradoxical chest wall movement was defined as the expansion of one compartment in the opposite direction in relation to the other that can occur during part-time or total time of the respiratory cycle [15]. In our study, the detailed analyses of inspiratory paradoxical chest movement include the phase shift angle and inspiratory paradox time between the pulmonary rib cage and abdomen. Subjects were classified with positive inspiratory paradoxical movement if both data were out of the normal range defined from healthy individuals.

The phase angle and inspiratory paradox time in our study were significantly different when we compared post-stroke subjects (general group) with healthy individuals. Furthermore, this observation was repeated when we assessed post-stroke subjects with more than three years of diagnosis. Regarding subjects with Parkinson’s disease (general group), the phase angle and inspiratory paradox time values were quite similar to healthy individuals. In relation to the inspiratory paradoxical movement, we found a positive inspiratory paradoxical movement in both groups of subjects (55% of the stroke subjects and 55.5% of Parkinson’s disease). In post-stroke and Parkinson’s disease subjects we also found a significant decrease in pulmonary rib cage volume when we compared subjects with positive versus negative inspiratory paradoxical movement. Moreover, post-stroke group subjects also showed significant differences in the volumes of the other compartments in the abdominal rib cage and abdominal. In line with our results related to diagnosis time, the data of inspiratory paradoxical movement and considering the results observed in the spirometry, we observed that post-stroke subjects independent of the allocation group showed low to mild restrictive respiratory disease, but with relative limitation in volumes during dynamic assessment.

It is important to make clear that, from the clinical point of view, the asynchronous ventilatory pattern found in the subjects of the present study should be seen and treated with care, since many of them may evolve with worsening of health-related quality of life due to pulmonary complications. This study it was important in detecting that neurological diseases, such as stroke and Parkinson’s disease, lead to modification in the breathing pattern, and that the breathing pattern seems to appear as an important respiratory alteration even in the absence of severe impairment of pulmonary function. Although our study presents some new findings on chest wall asynchrony and inspiratory paradoxical breathing movement in post-stroke and Parkinson’s disease subjects, some limitations must be reported. Post-stroke as well Parkinson’s disease comprises a combination of physiopathological factors in the central and peripheral nervous systems that influence the efficiency of ventilation. On the other hand, ventilation efficiency depends on chemoreceptor drive for breathing, respiratory muscles and airways; therefore, the clinical heterogeneity of both diseases hinders interpretation and data analyses. Nevertheless, the study power showed a large effect in Cohen’s analyses regarding the decrease of pulmonary rib cage volume.

## Conclusion

In conclusion, our study can contribute to better understanding of chest wall volumes, chest wall asynchrony and inspiratory paradoxical movement of breathing in post-stroke and Parkinson’s disease subjects with low to middle changes in spirometric values. In post-stroke subjects we found a decrease in the chest wall volume that is more evident in subjects with more than three years of diagnosis associated with impairment in breathing pattern. Moreover, inspiratory paradoxical movement was observed in half of the post-stroke subjects. In Parkinson’s disease, pulmonary rib cage volume was generally decreased and in patients with less than three years of diagnosis. Inspiratory paradoxical movement was also present in half of the Parkinson’s disease subjects, but no changes in breathing pattern were observed.
